# The GRAS transcription factor PtrPAT1 of *Poncirus trifoliata* functions in cold tolerance and modulates glycine betaine content by regulating the *BADH*-like gene

**DOI:** 10.1093/hr/uhae296

**Published:** 2024-10-23

**Authors:** Ruhong Ming, Tian Fang, Wei Ling, Jingjing Geng, Jing Qu, Yu Zhang, Jianhua Chen, Shaochang Yao, Liangbo Li, Ding Huang, Ji-Hong Liu

**Affiliations:** College of Pharmacy, Engineering Research Center of Innovative Traditional Chinese, Zhuang and Yao Materia Medica, Ministry of Education, Guangxi University of Chinese Medicine, Nanning 530200, China; National Key Laboratory for Germplasm Innovation and Utilization of Horticultural Crops, College of Horticulture and Forestry Sciences, Huazhong Agricultural University, Wuhan 430070, China; College of Pharmacy, Engineering Research Center of Innovative Traditional Chinese, Zhuang and Yao Materia Medica, Ministry of Education, Guangxi University of Chinese Medicine, Nanning 530200, China; College of Horticulture, Hebei Agricultural University, Baoding 071000, China; National Key Laboratory for Germplasm Innovation and Utilization of Horticultural Crops, College of Horticulture and Forestry Sciences, Huazhong Agricultural University, Wuhan 430070, China; Hubei Key Laboratory of Germplasm Innovation and Utilization of Fruit Trees, Research Institute of Fruit and Tea, Hubei Academy of Agricultural Sciences, Wuhan 430064, Hubei, China; College of Pharmacy, Engineering Research Center of Innovative Traditional Chinese, Zhuang and Yao Materia Medica, Ministry of Education, Guangxi University of Chinese Medicine, Nanning 530200, China; College of Pharmacy, Engineering Research Center of Innovative Traditional Chinese, Zhuang and Yao Materia Medica, Ministry of Education, Guangxi University of Chinese Medicine, Nanning 530200, China; College of Pharmacy, Engineering Research Center of Innovative Traditional Chinese, Zhuang and Yao Materia Medica, Ministry of Education, Guangxi University of Chinese Medicine, Nanning 530200, China; College of Pharmacy, Engineering Research Center of Innovative Traditional Chinese, Zhuang and Yao Materia Medica, Ministry of Education, Guangxi University of Chinese Medicine, Nanning 530200, China; National Key Laboratory for Germplasm Innovation and Utilization of Horticultural Crops, College of Horticulture and Forestry Sciences, Huazhong Agricultural University, Wuhan 430070, China

## Abstract

GRAS, termed after gibberellic acid insensitive (GAI), RGA (repressor of GA1), and SCR (scarecrow), is a plant-specific transcription factor crucial for plant development and stress response. However, understanding of the functions played by the GRAS members and their target genes in citrus is limited. In this study, we identified a cold stress-responsive GRAS gene from *Poncirus trifoliata*, designated as PtrPAT1, by yeast one-hybrid library screening using the promoter of *PtrBADH-l*, a betaine aldehyde dehydrogenase (BADH)-like gene. PtrPAT1, belonging to the PAT1 subfamily, was localized in the nucleus and plasma membrane, exhibited transactivation activity and showed a remarkable upregulation under cold stress. Overexpression of *PtrPAT1* elevated BADH activity, increased glycine betaine (GB) accumulation, and conferred enhanced cold tolerance in transgenic tobacco plants compared with wild type, while downregulation in trifoliate orange by virus-induced gene silencing (VIGS) resulted in opposite trends. Furthermore, the activities of two antioxidant enzymes, including peroxidase (POD) and superoxide dismutase (SOD), were significantly increased in the overexpression plants, but remarkably decreased in the VIGS line, consistent with accumulation patterns of the reactive oxygen species (ROSs). PtrPAT1 was demonstrated to interact with and activate the *PtrBADH-l* promoter through the putative PAT1-binding motif with the core sequence of TTTCATGT, indicating that *PtrBADH-l* is a target gene of PtrPAT1. Taken together, these results demonstrate that PtrPAT1 positively affects cold tolerance through the regulation of GB biosynthesis by modulating *PtrBADH-l* expression.

## Introduction

Environmental factors, such as high or low temperature, drought, and salt, shape the spatial distribution of plant species in their natural habitats [1]. Among these, cold stress adversely affects agricultural crop productivity and global food security [2, 3]. When plants experience unfavorable environmental temperature conditions, many sophisticated mechanisms occur to help plants adapt to the change, ranging from signal perception to transmission, and then to a series of physiological and biochemical responses. Suitable low temperature conditions without freezing, known as cold acclimation, can enable plants to mitigate the detrimental effects of cold exposure and thereby enhance their tolerance to cold [4]. Thus, elucidating the changes induced by cold acclimation will offer insights into understanding the mechanisms underlying plant adaptation to cold stress.

On exposure to cold environments, plants typically promote the biosynthesis and accumulation of specific small molecule metabolites such as sugar alcohols, proline, and glycine betaine (GB). These compatible substances, known for their high solubility and nontoxic characteristics, are crucial for maintaining cellular osmotic balance. GB, a well-recognized osmoprotectant, accumulates in response to dehydration stress and is a key compound in this process [5–7]. Recent research indicates that low temperatures can also trigger an increase in GB content in plant cells, leading to various benefits such as reducing cell osmotic potential, preserving turgor pressure, minimizing plasma membrane damage, and enhancing stress resistance [8, 9]. GB functions as a protective agent by serving as a molecular chaperone to help protein folding and safeguard the structural integrity of biological macromolecules. In addition, it contributes to safeguarding photosynthesis, increasing antioxidant ability, and influencing stomatal characteristics to enhance abiotic stress tolerance [10–12]. To mitigate plant stress damage and enhance plant stress resistance, GB has been extensively used as an exogenous growth regulator in tomato [13], wheat [14], and barley [15].

Transcription factors (TFs) can precisely control downstream gene expression by binding to the *cis*-elements in promoters, and their regulatory functions play an important role in current plant research. In response to adverse environmental stimuli, numerous TFs (bHLH, MYB, NAC, ERF, etc.) are typically activated or degraded, which in turn governs the synthesis or decomposition of downstream metabolites. For example, Han *et al*. [16] demonstrated that *FvICE1* functioned as a positive regulator of cold and drought resistance by overexpression and CRISPR/Cas9 technologies in strawberry. As a class of plant-specific TF families, the GRAS TFs generally comprise 400–800 amino acid residues and are characterized by a notably conserved GRAS domain at the C-terminal and a highly variable N-terminal region. The GRAS domain is composed of 390 amino acids and consists of five motifs arranged in a specific sequence: leucine heptad repeat I (LHR I), VHIID, LHR II, PFYRE, and SAW. The plant-specific GRAS proteins are segmented into eight distinct branches (PAT1, LISCL, DELLA, SCL3, SHR, SCR, LS, and HAM) in rice and Arabidopsis, while they are segregated into 11 distinct categories in sweet orange [17, 18]. To date, GRAS TFs perform diverse molecular functions and regulate multiple biological processes, including organ development [19], phytohormone signaling pathways [20], and phytochrome signal transduction [21]. Moreover, emerging studies have suggested that the GRAS TF members are widely involved in plant response to abiotic stress [22]. For example, SlGRAS40 overexpression significantly increased drought resistance and salt tolerance, which can be attributed to the regulation of auxin and gibberellin (GA) homeostasis [23], while only SlGRAS4 was markedly induced after low-temperature treatment and directly regulated many cold-responsive genes to improve the cold tolerance of tomato [24]. The GRAS TF VaPAT1 plays a role in regulating plant stress resistance, and the cold-responsive molecular mechanism was further illustrated in grape [25, 26]. However, the relationship between GRAS family members and cold tolerance of trifoliate orange is still rarely reported.

Citrus species are evergreen trees belonging to the Rutaceae family, which thrive in warm, humid environments, and are naturally found between the latitudes of 40°S and 40°N in tropical and subtropical regions [27]. The geographic limitations on their growth restrict the rapid development of the citrus industry. To mitigate the damage caused by low temperature to commercial citrus varieties, cold-resistant trifoliate orange (*Poncirus trifoliata* L. Raf.) is commonly used as the rootstock, mainly because it can endure temperature as low as −26°C when fully cold-acclimated, making it a potential key resource for citrus genetic improvement [28]. Based on a transcriptome analysis of trifoliate orange after cold treatment, the *PtrBADH-l* gene, a betaine aldehyde dehydrogenase (BADH)-like gene, that encodes the limited enzyme of GB biosynthesis was identified [29]. Subsequent functional verification showed that this gene responded strongly to low temperature, and PtrMYC2 was shown to bind to the *PtrBADH-l* promoter as a positive regulator that mediates cold tolerance by promoting GB accumulation in citrus plants [30]. Nevertheless, further studies are needed to fully understand and explore other regulating factors of *PtrBADH-l*, especially their specific role in GB biosynthesis and stress tolerance.

In this study, we identified the function of PtrPAT1 in low-temperature tolerance associated with GB accumulation. Our results demonstrate that PtrPAT1 acts as a positive regulator of cold tolerance partly due to its role in modulating GB content by the regulation of BADH-l protein. Thus, *PtrBADH-l* is a potential target gene of PtrPAT1. Moreover, PtrPAT1 may modulate reactive oxygen species (ROS) scavenging by increasing the activity of ROS scavenging enzymes, thus enhancing cold tolerance. Taken together, this study reveals that PtrPAT1 enhances the tolerance of trifoliate orange to low-temperature by regulating GB biosynthesis—a new mechanism for responding to cold stress.

## Results

### Identification of *PtrPAT1* and sequence analysis

*PtrBADH-l* is associated with GB accumulation under cold in citrus plants. To understand the regulatory mechanism of GB biosynthesis in response to cold in citrus, a yeast one-hybrid (Y1H) screening of the cDNA library of trifoliate orange was used to identify several positive clones, using the promoter of the cold-responsive target gene *PtrBADH-l* as bait [30]. By sequencing and BLASTP alignment using the *Citrus* Pan-genome to Breeding Database (CPBD) (http://citrus.hzau.edu.cn/) and the *Arabidopsis* Information Resource (TAIR) (https://www.arabidopsis.org/), a previously unidentified potential TF was revealed—the GRAS member PtrPAT1 (Pt1g013090, *P. trifoliata* scarecrow-like TF PAT1).

**Figure 1:**
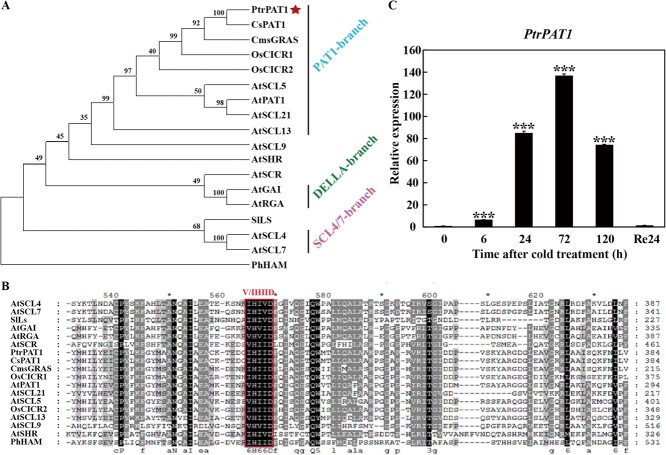
Characterization analysis of the cold-responsive gene PtrPAT1. (A, B) Phylogenetic analysis (A) and amino acid sequence alignment (B) of PtrPAT1 with other GRAS proteins. The red solid pentagram represents PtrPAT1, and the red box marks the conserved V/I HIID motif. PAT1 branch proteins (AtPAT1, At5g48150; OsCIGR1, AAL61820.1; OsCIGR2, Q8GVE1.1; CmsGRAS, JF440647.1; AtSCL5, At1g50600; AtSCL13, At4g17230; AtSCL21, At2g04890), DELLA branch proteins (AtRGA, At2g01570; AtGAI, At1g14920), SCL4/7 branch proteins (AtSCL4, At5g66770; AtSCL7, At3g50650), and other branch proteins (AtSCL9, At2g37650; AtSHR, At4g37650; AtSCR, At3g54220; SlLs, AAD05242.1; and PhHAM, AAM90848.1). (C) Relative expression level of *PtrPAT1* under low temperature.

The *PtrPAT1* gene contained a 1743-bp full-length open reading frame and was projected to transcribe 580 amino acids, with an expected molecular weight of 6.40 kD. Additional predicted attributes include an isoelectric point of 6.12, an instability index of 54.15, and a grand average of hydropathicity of −0.313. Phylogenetic analysis of PtrPAT1 and other PAT1 proteins associated with abiotic stresses from selected plant species, including *Citrus sinensis*, *C. medica*, *Oryza sativa*, *Arabidopsis thaliana*, *Solanum lycopersicum*, and *Petunia hybrid*, indicated that it belonged to the PAT1 subfamily and was most closely related to CsPAT1 and CmsGRAS from two citrus relatives (Fig. 1A). Multiple alignment analysis revealed that PtrPAT1 contains a highly conserved motif of GRAS domains in the C-terminal region—V/IHIID domain (Fig. 1B). The expression pattern of *PtrPAT1* showed that it was strongly induced by cold stress, specially peaked at 72 h (approximately 140-fold of that at 0 h) (Fig. 1C), and a similar tendency for *PtrBADH-l* confirmed by a previous report [30]. These results suggest that *PtrPAT1* is a cold-responsive gene.

### PtrPAT1 localizes in both nucleus and plasma membrane and exhibits transcriptional activation activity

**Figure 2:**
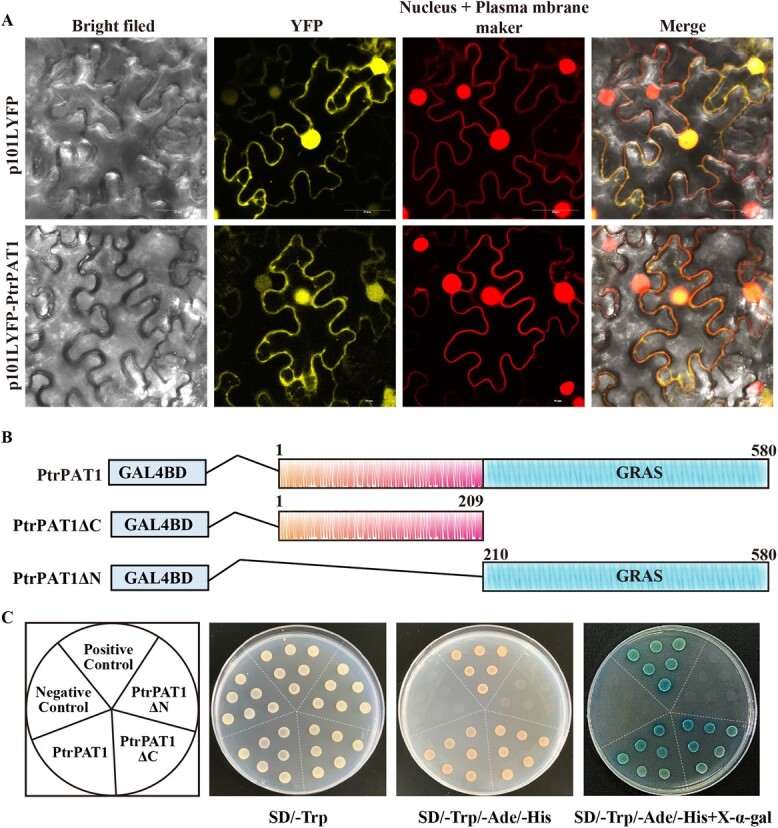
Subcellular localization and transcriptional activity assays of PtrPAT1. (A) PtrPAT1 was in-frame fused with YFP and transiently expressed in tobacco leaf epidermal cells. 101YFP was used as the control, and mCherry and CBL1n-OFP were used as markers for the nucleus and plasma membrane, respectively. Bar, 25 μm. (B) Schematic of various vector constructs used for transcriptional activity examinations of PtrPAT1. PtrPAT1ΔC and PtrPAT1ΔN symbolize the elimination of the C and N terminals, respectively. (C) Proliferation of yeast strain AH109 transformed constructs was monitored on selective synthetic dextrose media. The positive and negative controls were pGBKT7–53+pGADT7-T and pGBKT7, respectively.

To elucidate the function of PtrPAT1, the subcellular localization of PtrPAT1 was examined by transiently transfecting the PtrPAT1-YFP fusion protein into leaf epidermal cells of *Nicotiana benthamiana* (tobacco). In cells transfected with the control vector, the YFP signal was uniformly distributed throughout the entire cell. Conversely, in cells expressing the PtrPAT1-YFP fusion protein, yellow fluorescence was evident in both nucleus and plasma membrane. This observation was corroborated by the colocalization of yellow fluorescence with the orange fluorescence signal of the nucleus and plasma membrane marker proteins (Fig. 2A), indicating the presence of PtrPAT1 in the nucleus and plasma membrane. In addition, the transcriptional activity of PtrPAT1 was assessed in yeast. PtrPAT1 was divided into two parts based on the N-terminal and conserved C-terminal regions (Fig. 2B). Full-length PtrPAT1 and truncated forms PtrPAT1ΔC (aa: 1–209) and PtrPAT1ΔN (aa: 210–580) were each fused with the GAL4 DNA-binding domain. PtrPAT1 and PtrPAT1-N term facilitated yeast growth on SD/−T/−A/-H plates with X-gal activity, whereas PtrPAT1-C term did not exhibit growth on these plates (Fig. 2C). These findings provide evidence for PtrPAT1 functioning as a transcriptional activator.

### PtrPAT1 overexpression improves cold tolerance in transgenic plants

**Figure 3:**
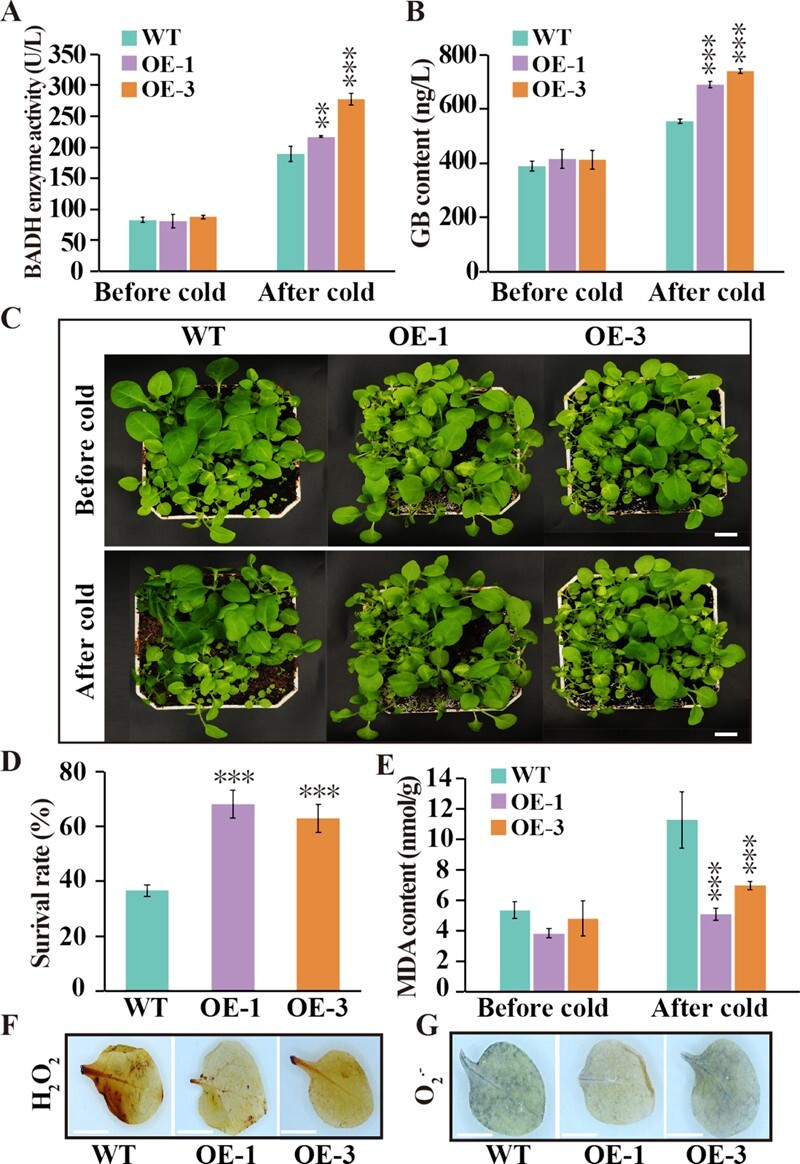
PtrPAT1 overexpression amplifies cold tolerance in tobacco. (A, B) BADH activity (A) and GB content (B) of WT and transgenic plants (OE-1 and OE-3) were compared before and after exposure to cold conditions (0°C for 5 h). (C) Morphological comparison of WT and OE lines (1 and 3) before and after cold exposure. Bar, 1 cm. (D) Survival rate of WT and OE lines (1 and 3) after cold and recovery conditions (25°C for 24 h). (E) MDA levels of WT and OE lines (1 and 3) with and without exposure to cold. (F, G) *In situ* histochemical staining used for detecting H_2_O_2_ accumulation (F) and O_2_^•−^ (G) in WT and OE lines (1 and 3) after cold exposure. Bar, 1 cm. Data are means (±SE), *n* = 3. Under identical growth conditions, asterisks symbolize noteworthy differences between OE lines (1 and 3) and WT control. *P* ≤ 0.001.

Considering the established role of PAT1 in grapevine in mediating cold tolerance and the above identification of PtrPAT1 as an upstream modulator of *PtrBADH-1*, it was postulated that PtrPAT1 participates in cold tolerance mechanisms by modulating GB biosynthesis. For this purpose, transgenic tobacco lines overexpressing PtrPAT1 were derived via *Agrobacterium*-mediated transformation (Fig. S1), and two transgenic lines (#1 and #3) showing elevated transcript levels were chosen for cold tolerance assays (Fig. S2). The two transgenic lines exhibited slightly elevated BADH activity and GB content compared with wild type (WT) plants under normal conditions. After exposure to cold treatment, BADH activity and GB content slightly increased in WT plants, while a more pronounced increase was observed in transgenic lines, resulting in a significant difference between the two groups (Fig. 3A, B). Under optimal growth conditions, there were no obvious morphological differences between WT and transgenic plants. However, when all plants underwent cold treatment (0°C for 5 h), WT plants exhibited a pronounced water-soaked appearance compared with transgenic plants (Fig. 3C). After a 1-day recovery period at room temperature, the survival rates of transgenic plants (68.1% and 62.8%) were significantly higher than that (36.6%) of WT plants (Fig. 3D). Cold stress induces ROS production leading to cellular oxidative injury, and the buildup of MDA, a byproduct of lipid oxidation by ROS, serves as a marker for membrane oxidative damage. Remarkably, the leaves of OE lines exposed to cold exhibited reduced MDA levels compared with WT, suggesting that the upregulation of *PtrPAT1* leads to lower oxidative injury (Fig. 3E). *In situ* histochemical staining with DAB (for H_2_O_2_) and NBT (for O_2_^−^) was used to qualitatively detect ROS production and accumulation between WT and transgenic plants. Transgenic plants accumulated profoundly lower levels of ROS after exposure to cold, as evidenced by the more intense and pronounced staining in WT leaves compared with transgenic plants (Fig. 3F, G). Taken together, these findings demonstrate that the heterologous expression of PtrPAT1 fostered an increase in GB accumulation and significantly improved resistance to low temperatures, accompanied by a reduction in membrane damage and the elimination of ROS.

### Silencing of *PtrPAT1* results in hypersensitivity to cold stress

**Figure 4:**
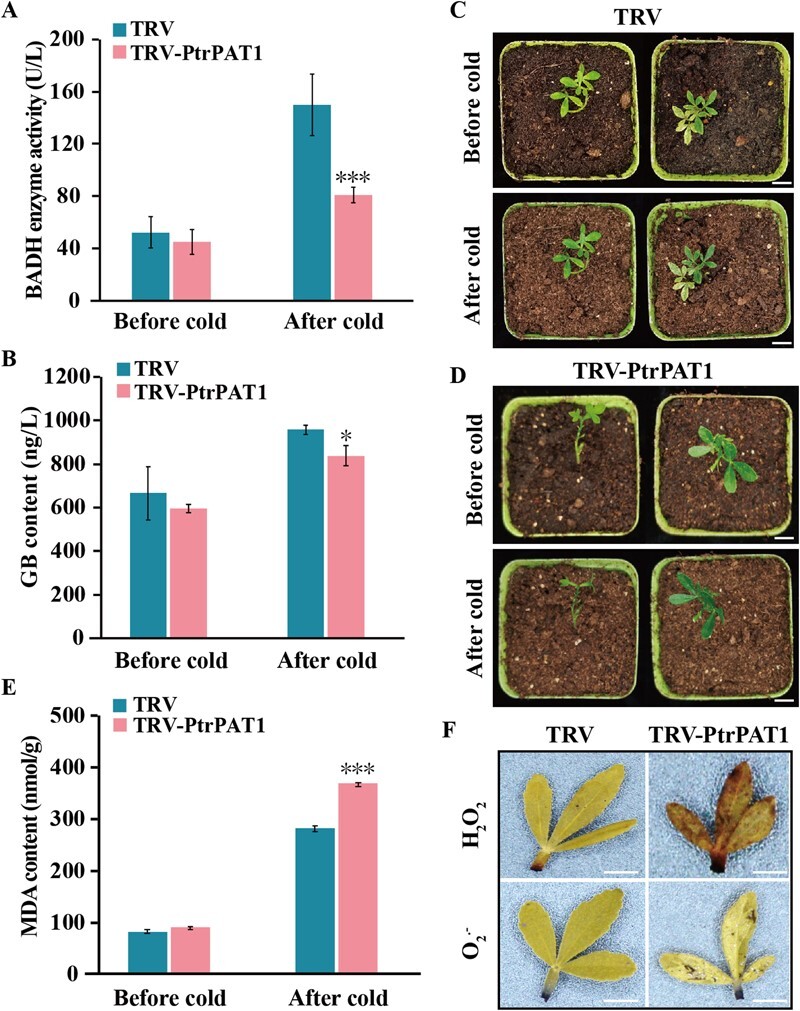
Silencing of PtrPAT1 significantly enhanced the cold sensitivity of *P. trifoliata*. (A, B) BADH activity (A) and GB content (B) in TRV control and TRV-PtrPAT1 seedlings with and without cold treatment (0°C for 48 h). (C, D) Phenotype of TRV control (C) and TRV-PtrPAT1 (D) plants taken before and after exposure to cold stress. Bar, 1 cm. (E) The MDA level in the TRV-PtrPAT1 lines and TRV control ascertained before and after cold treatment. (F) *In situ* histochemical staining for detecting the accumulation of H_2_O_2_ and O_2_^•−^ in TRV control and TRV-PtrPAT1 plants subjected to cold treatment. Bar, 1 cm. Error bars denote ±SE, *n* = 3. In identical growth environments, asterisks indicate marked deviations between the VIGS (TRV-PtrPAT1) line and the TRV control.

To investigate the role of PtrPAT1 in modulating cold tolerance and GB biosynthesis, PtrPAT1 was suppressed using virus-induced gene silencing (VIGS) in *P. trifoliata* (Fig. S3). This knockdown resulted in a simultaneous decrease in *PtrBADH-l* expression (Fig. S4). The VIGS plants demonstrated decreased BADH activity and GB content compared with control plants, with the discrepancy becoming more noticeable and statistically relevant under cold treatment (Fig. 4A, B). Under normal growth conditions, no significant morphological differences were observed between TRV control plants and VIGS lines. When exposed to low-temperature treatment (0°C for 48 h), the VIGS line exhibited a significant deficiency in tolerance to cold conditions, accompanied by severe leaf wilting and curling compared with control plants (Fig. 4C, D). Consistent with this result, MDA levels were significantly higher in VIGS plants than in control plants (Fig. 4E). Besides, the staining experiments using DAB and NBT demonstrated that PtrPAT1-silenced plants exhibited higher levels of H_2_O_2_ and O_2_^−^, compared with control plants. Thus, under cold stress, membrane injuries were more severe in VIGS plants than in control plants. These findings were supported by *in situ* histochemical staining results (Fig. 4F). Collectively, these data demonstrate that *PtrPAT1* knockdown severely impaired GB accumulation and accelerated cold sensitivity, implying that PtrPAT1 plays a role in cold tolerance by regulating GB biosynthesis.

### Alteration of antioxidant enzyme activities in transgenic and silenced plants

**Figure 5:**
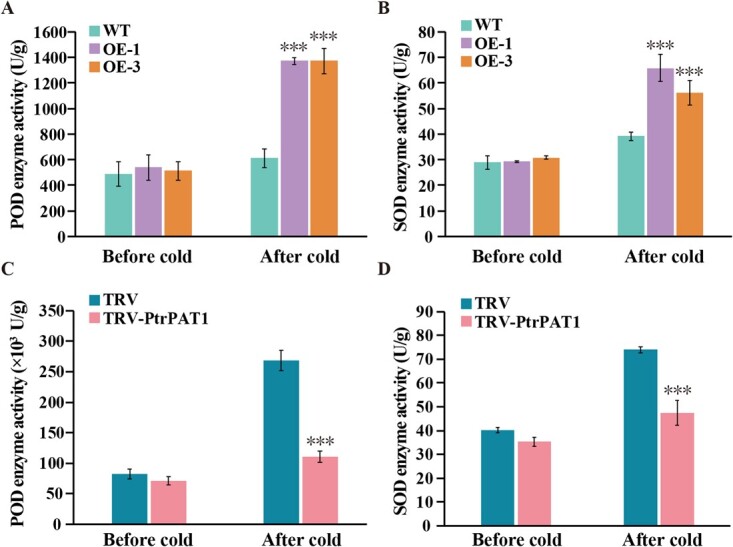
Assessment of antioxidant enzyme functions in tobacco plants and silenced trifoliate orange. (A, B) Activity variations of POD (A) and SOD (B) in tobacco WT and transgenic lines (OE-1, OE-3) before and after low temperature exposure (0°C for 5 h). (C, D) Changes in POD (C) and SOD (D) activities in TRV and TRV-PtrPAT1 silenced trifoliate orange plants before and after exposure to cold (0°C for 48 h). Error bars denote ±SE, *n* = 3. The asterisks denote statistically significant differences between control and OE or silenced lines under the same growth conditions (*P* ≤ 0.001).

Considering the DAB and NBT staining results for transgenic tobacco and PtrPAT1-silenced trifoliate orange under low-temperature conditions as mentioned above, the investigation of the activities of two key antioxidant enzymes, peroxidase (POD) and superoxide dismutase (SOD) was motivated by their ROS-scavenging role. Under normal conditions, the two enzyme activities were marginally higher in transgenic tobacco lines compared with WT (Fig. 5A, B); however, a substantial increase in enzyme activities was observed in transgenic plants relative to WT under cold condition. In contrast, the enzyme activities in PtrPAT1-silenced plants (pTRV-PtrPAT1) were significantly decreased compared with WT both before and after cold treatment (Fig. 5C, D). These findings suggest that PtrPAT1 also responds to low-temperature stress by regulating the transcriptional expression of *POD* and *SOD* genes to control ROS accumulation.

### PtrPAT1 binds to and activates the promoter of *PtrBADH-l*

**Figure 6:**
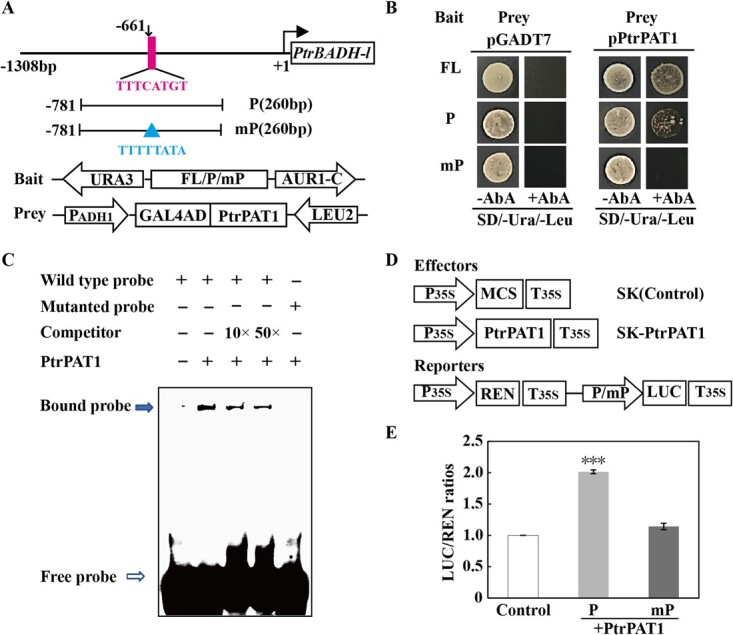
PtrPAT1 exhibits specific affinity for the *PtrBADH-l* promoter and functions as a transcriptional activator. (A) *PtrBADH-l* promoter and prey and bait vectors used in the Y1H assay. The pink stripe represents the putative PAT1-binding motif in the P fragment, while the blue triangle represents a mutation at the corresponding binding site in the mP fragment. (B) Growth of yeast Y1H-Gold involving various bait and prey combinations on selective media (SD/-Ura/−Leu) supplemented with or without AbA. (C) EMSA demonstrating direct and specific attachment between the TTTCATGT motif and PtrPAT1. The closed and open arrows represent the bound DNA–protein complex and free probe, respectively. −, absence, +, presence. (D) Effector and reporter constructs used for dual luciferase assays. (E) Results of PtrPAT1 dual luciferase assays using different promoter fragments in the transient assay. In the absence of effectors, the LUC/REN ratio in the control group was normalized to 1. Values are mean ± SE (*n* = 3). Asterisks indicate significant difference between each other (*P* < 0.001).

To identify the molecular mechanism of PtrPAT1 in regulating *PtrBADH-l* response to cold stress, Y1H, electrophoretic mobility shift assay (EMSA), and luciferase (LUC) assays were performed. Our previous research revealed that *PtrBADH-l* upregulation was induced by cold, with the 573-bp promoter segment (−786 to −213 bp) playing a crucial role [30]. Thus, this key fragment was further analyzed for mining the *cis*-acting element recognized by PtrPAT1. Given that no reports have pinpointed the binding sites of PAT1 subfamily members, we found a putative PtrPAT1-binding motif (TTTCATGT, −661 to −668 bp) in the promoter of *PtrBADH-l*, which was hinted as a PtrPAT1-binding site, according to the ChIP-Seq data of the cold-responsive gene SlGRAS4, only one TF of the GRAS family was induced by low temperature in tomato [24]. Therefore, the Y1H assay demonstrated the robust growth of yeast cells transformed with prey and baits comprising FL and P fragments containing the TTTCATGT motif on selective media. However, upon mutation of the potential motif within the P fragment, yeast cell growth was abrogated (Fig. 6A, B). Subsequently, EMSA was performed to ascertain the potential direct interaction between PtrPAT1 and the TTTCATGT motif. Incubation of HIS-PtrPAT1 protein with labeled probes induced a noticeable band shift, while the inclusion of unlabeled competitor DNA dose-dependently mitigated this electrophoretic mobility shift. Notably, mutation of the motif within the probes entirely abolished the observed band shift (Fig. 6C), thus affirming the direct and specific binding of PtrPAT1 to the TTTCATGT motif within the P regions. Quantitative dual LUC assay demonstrated that PtrPAT1 activated *PtrBADH-l* by interacting with the motif within the promoter, while mutation of this motif impaired the activation (Fig. 6D, G). Collectively, these results demonstrate that PtrPAT1 acts as a transcriptional activator of *PtrBADH-l* by interacting with the TTTCATGT motif.

## Discussion

Citrus fruits are favored worldwide for their distinct taste and rich nutritional profile. In 2021, citrus fruits ranked as the second most produced fruit, yielding 161.8 million tons across over 10.2 million ha of land [31]. The effect of climate change has resulted in more frequent and intense cold attacks, causing substantial agricultural damage by reducing crop yields and fruit quality. The response to cold stress in citrus fruits has been the subject of extensive research because of the widespread use of cold storage to lengthen the post-harvest period so that fruits remain edible [27, 32]. Moreover, numerous studies have focused on evaluating the resilience of citrus rootstocks because of their cold tolerance. As an elite cold-hardy plant, *P. trifoliata* L. Raf. (also known as *C. trifoliata* L.) is an ideal genetic resource for the identification of cold-tolerance associated genes and citrus rootstocks to improve cold tolerance in citrus. Hence, numerous studies have been performed to identify and validate cold tolerance genes in trifoliate orange [33, 34]. Previously, we identified and characterized a key enzyme gene, *PtrBADH-l*, which is involved in GB biosynthesis and exhibits a strong response to cold stress, and the mechanism by which a transcriptional regulatory module responds to low temperature has been systematically revealed [30]. However, one metabolite is usually targeted by various regulatory factors at the transcriptional regulatory level. In this study, we identified a new transcription factor, PtrPAT1, based on Y1H library screening using the promoter of *PtrBADH-l*, and resolved another regulatory module PtrPAT1-PtrBADH-l of GB biosynthesis in response to low temperature. The present study provides new insights into the role of the PtrPAT1 gene in mediating cold tolerance, expands our understanding of the regulatory network associated with GB metabolism, and provides genetic resources for engineering cold tolerance in plants.

As genomic sequencing technology has advanced, TF families that respond to various abiotic stresses have been identified in many plants. For instance, PbERF3 was activated in response to drought and forms an interaction with PbHafC1a to controls the expression of genes related to ABA biosynthesis and H_2_O_2_ [35]; *ApbHLH47* and *ApbHLH109* were significantly downregulated under salinity, salicylic acid and 6-benzylaminopurine treatments, implying they have essential functions in response to abiotic stresses and plant development [36]; and OsMYB30 was negatively regulated of the *BMY* genes by interacting with OsJAZ9 to control the starch breakdown and the content of maltose in response to cold [37]. The GRAS family members are involved in a range of physiological and developmental processes, including not only plant growth and differentiation but also signaling in response to phytohormones, forming synergistic relationships to help plants adapt to environmental cues [38]. Several candidate genes of the PAT1 subfamily, a member branch of GRAS, have not yet been functionally characterized, although they have been isolated in few fruit trees. Zhang *et al*. [18] found that sweet orange *GRAS7*, the closest to *AtPAT1*, was induced by Pi-deficiency, GA treatment, and salt stress. VaPAT1 is a positive transcriptional regulator in grapevine that plays a role in abiotic stress responses [25]. Nevertheless, research investigating the function and molecular mechanism of PAT1 from *P. trifoliata* in response to cold stress is lacking. Hence, we silenced PtrPAT1 expression in trifoliate orange seedlings and heterologously overexpressed PtrPAT1 in tobacco leaves. A series of physiological experiments demonstrated that PtrPAT1 positively regulates BADH activity and GB accumulation upon cold condition, as well as ROS scavenging.

Although GRAS proteins play a role in transcriptional regulation and are considered to bind directly to DNA, most of them indirectly regulate the expression of downstream target genes by forming complexes with other TFs or proteins. For example, several GRAS members, particularly DELLA proteins lacking the DNA-binding domain, usually combined with indeterminate domain (IDD) proteins to regulate plant growth and development [39]. The mitotic GRAS-type AtSCL28 formed a heterodimer with the AP2-type gene *AtSMOS1*, which led to direct transcriptional activation of the *SMR* family genes to balance cell size and cell number in developing organs of plants [40]. In addition, some DELLA genes exhibited notable responsiveness to various abiotic stresses by increasing the expression levels of genes that encode ROS-detoxifying enzymes, thus reducing ROS levels, delaying cell death, and enhancing resistance [41]. Recent studies have uncovered the direct regulatory mechanism of GRAS TFs in tomato. Liu *et al*. [42] showed that SlGRAS4 can act as a transcription factor to regulate fruit ripening by directly binding to the promoters of ethylene biosynthesis genes *SlACO1* and *SlACO3* to activate their transcription. Besides, this team verified that under cold treatment, SlGRAS4 could bind and activate the promoters of target genes involved in antioxidant capacity (*SlPOD*, *SlAPX*, *SlGPX*, *SlLOX*), calcium signaling (*calcium-transporting ATPase*, *calmodulin-binding protein*), photosynthesis ability (*Rubisco*), and energy metabolism (*SlPECK*, *SlMDH*), as well as *SlCBF1*, *SlCBF2*, and *SlCBF3* [24], suggesting that SlGRAS4 enhances cold tolerance by both *CBF-independent* and *ICE1-CBF* pathways in tomato.

However, to the best of our knowledge, the regulatory mechanisms of PAT1 are still poorly understood and have only been reported in individual plants. For instance, the interaction between PAT1 and ERF115 leads to the formation of the ERF115–PAT1 complex, which is involved in regulating the regenerative capacity of the root meristem and coordinating a wound-induced response in root-knot nematode induced galls [43]. In *Vitis amurensis*, PAT1 indirectly enhances the expression of *LOX3* and stimulates jasmonate (JA) synthesis through its interaction with IDD3 to promote cold tolerance [26]. Recently, Li *et al*. [44] found that CsPAT1 facilitated lignin accumulation by antagonistic interaction with CsWRKY33, rather than by the direct regulation of downstream lignin biosynthesis genes, which were the targets of CsWRKY33 in tea plants. However, according to this study, PtrPAT1 can directly bind to the specific motif (TTTCATGT) on the promoter of the GB biosynthase gene *PtrBADH-l*, and then activate its expression to control GB accumulation, which was supported by molecular experiments such as Y1H, EMSA, and LUC, thereby enhancing its cold tolerance. This mechanism shares similarities with the function of SlGRAS4 in tomato, although GRAS4 does not share homology with PAT1 in tomato, implying that the different families of GRAS genes might have similar DNA-binding preferences and biological functions [24]. In addition, the silencing of PAT1 in *P. trifoliata* resulted in a significant reduction in gene expression and enzyme activities of POD and SOD (Fig. S4; Fig. 5). However, further experimental validation is required to elucidate the direct specific mechanism between PtrPAT1 and PtrPOD, as well as PtrSOD, in response to low temperature.

Studies on the transcriptional regulation of GB biosynthesis have been relatively shallow. GB biosynthesis in plants is subject to modulation by diverse cues including abiotic stressors and hormonal stimuli [45]. As part of the GRAS gene family, the PAT1 protein is recognized as a positive factor in the phyA-dependent light signaling pathway and contributes to hormonal signal transduction [46]. When plants experience low temperatures, the gibberellic acid (GA) endogenous levels decrease. Consequently, DELLA proteins, which compete with the JAZ proteins of MYC2, are released. This release promotes the binding of MYC2 to the G-box elements on the promoter of downstream cold-responsive genes, thereby enhancing cold tolerance [47, 48]. In combination with our previous results that JA-induced PtrMYC2 positively regulates GB biosynthesis in response to cold, it is pertinent to inquire whether PAT1 participates in the GA signaling pathway and indirectly modulates MYC2 response to low temperature by the interaction between JAZ proteins, consequently fostering GB accumulation and enhancing plant resistance. Hence, our forthcoming research endeavors will focus on investigating the regulatory framework of the GB biosynthesis pathway by integrating PAT1-mediated environmental cues and hormonal signaling pathways.

**Figure 7:**
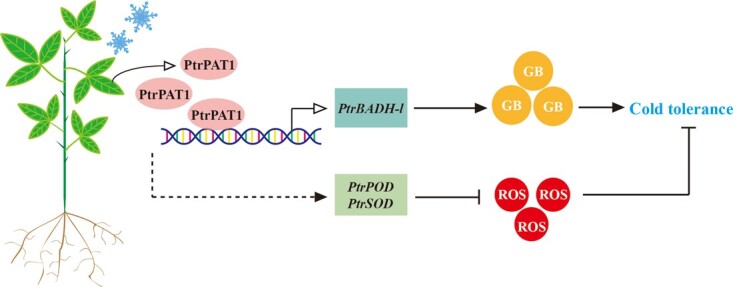
A simplified model illustrating the regulation of PtrPAT1 mediated under cold stress in trifoliate orange. Exposure to cold induces PtrPAT1, which transactivates *PtrBADH-l* by directly binding to its promoter, thus enhancing GB accumulation. Moreover, the cold-induced PtrPAT1 could regulate the expression of *PtrPOD* and *PtrSOD* either directly or indirectly, thus facilitating ROS removal. This, along with the two independent pathways, ultimately enhances the cold tolerance of *P. trifoliata*. The solid lines indicate a direct role, and dashed lines indicate an indirect role. Whether PtrPAT1 regulates the transcription of *PtrPOD* and *PtrSOD* in other ways needs further investigation.

In sum, our study demonstrated that PtrPAT1 was an important regulator of cold tolerance in citrus, leading to the proposition of a hypothetical working model (Fig. 7). Upon low temperature, PtrPAT1 exhibits notable induction and consequently facilitates the activation of the *PtrBADH-l* promoter by directly binding to the specific motif TTTCATGT, thereby stimulating GB biosynthesis and accumulation. In addition, PtrPAT1 may exert direct or indirect control over the expression of *PtrPOD* and *PtrSOD*, leading to increased POD and SOD levels, thereby partially modulating ROS clearance, ultimately enhancing the overall cold tolerance of trifoliate orange. The results put forward the idea that PAT1-BADH-l module is involved in the regulation of plant cold tolerance, which not only offers a fresh perspective on the regulatory network of cold tolerance in citrus plants but also provides valuable insights into GB biosynthesis and the GRAS gene family in plants.

## Materials and methods

### Plant materials and growth conditions

The trifoliate orange seedlings for gene cloning and low-temperature treatment were adopted based on a previous report [30]. The leaves were obtained at 4°C for 0, 6, 24, 72, 120 h and transferred to ambient conditions for 24 h. Foliage samples, obtained from a minimum of three biological replicates, were promptly immersed in liquid nitrogen for freezing, and then maintained at −80°C for further analysis. Concurrently, identical growth conditions were established for the cultivation of *Nicotiana benthamiana* (tobacco). After tobacco plants reached the developmental stage of five leaf pairs, they were used for subcellular localization analysis and dual LUC assays.

### RNA extraction and gene expression analysis

RNA was extracted from samples using a commercial kit (AiDLab, China) according to the manufacturer’s protocol. The RevertAid First-Strand cDNA Synthesis Kit (Thermo Fisher Scientific, USA) was used for cDNA synthesis. In subsequent steps, a quantitative real-time polymerase chain reaction (qRT-PCR) was performed using a RT-PCR system (CFX96 Touch System, USA) with AceQ SYBR Green Master Mix (Vazyme, China). Each sample underwent quadruplicate analysis, with the transcription levels of target genes calculated by the 2^−ΔΔCt^ method [49]. *β-Actin* in *P. trifoliata* and *Ubiquitin* in tobacco were used as reference genes for normalization. Details of the primer sequences used are presented in Table S1.

**Table S1:** 

### Gene isolation and bioinformatics analysis

The coding sequences (CDSs) of PtrPAT1 were amplified using designated primers as specified in Table S1. ExPASy was used to assess the molecular weight, isoelectric point, instability index, and grand average of hydropathicity of PtrPAT1. Amino acid sequence alignments across species were performed using ClustalW and GeneDoc software. Phylogenetic analysis was performed using the neighbor joining method in the MEGA X application. Protein sequences of the other plants were downloaded from the NCBI database.

### Subcellular localization analysis

The stop codon-deficient CDS fragment of PtrPAT1 was amplified, and the PCR-amplified product was then ligated into the p101LYFP vector, following its digestion with EcoRI and BamHI restriction enzymes. The chimeric plasmids p35S-PtrPAT1-YFP and p35S-YFP (used as a negative control) were cotransfected with CBL1n-OFP and mCherry, serving as markers for plasma membrane and nucleus, respectively, into tobacco leaves using *Agrobacterium tumefaciens* GV3101. After infiltration, plants were cultivated in a growth incubator for 2–3 days before visualizing YFP and OFP fluorescence under a confocal laser scanning microscope (Leica TCSSP8, Germany).

### Transcriptional activation analysis

CDSs of PtrPAT1 and its N-terminal and C-terminal truncated fragments were individually integrated into the pGBKT7 vector, which was digested with EcoRI and SalI restriction enzymes. The resulting constructs were then introduced into the yeast strain AH109 carrying a MEL1 reporter plasmid, followed by plating on selective media including SD/−Trp and SD/−Trp/−Ade/−His either with or without 4 mg/mL X-α-gal (Sigma-Aldrich). The pGBKT7–53 + pGADT7-T vector was the positive control, while the empty pGBKT7 vector was the negative control.

### Plasmid construction and plant transformation

The CDS of PtrPAT1 was amplified and integrated into the pH7WG2D vector, containing green fluorescence protein, by the gateway method. The TRV-based pTRV1/2 vectors were used for virus-induced gene silencing (VIGS). The construction of the pTRV-PtrPAT1 vector involved PCR amplification of a fragment of the PtrPAT1 open reading frame, approximately 338 bp in length, followed by its insertion into the pTRV2 vector. The pTRV2 vector, without any modifications, was used as a control. All modified vectors were subsequently inoculated into the *A. tumefaciens* strain GV3101. The OE vector was used to transform tobacco by the leaf disk method [50]. VIGS was performed by infiltrating the leaves of 30-day-old *P. trifoliata* seedlings with a bacterial mixture containing pTRV1 and pTRV2-PtrPAT1 in a 1:1 ratio [51]. Hygromycin-resistant OE plants were confirmed using a LUYOR-3415RG double fluorescent protein observation lamp to detect green fluorescence, followed by PCR and qRT-PCR analyses. T_2_ homozygous transgenic tobacco plants were used for subsequent experiments. Molecular characterization, including PCR and qRT-PCR analyses, was performed on the kanamycin-resistant VIGS seedlings. The primer sequences are presented in Table S1.

### Yeast one-hybrid (Y1H) assay

The full-length (FL) promoter of *PtrBADH-l*, as well as its fragmented (P) and mutated (mP) versions containing a putative PtrPAT1-binding motif, TTTCATGT, were amplified and inserted into the pAbAi vector as bait constructs. The bait was used for conducting Y1H screening against a cDNA library of trifoliate orange as described previously [30]. Subsequently, these bait plasmids were introduced into the Y1H Gold yeast strain by transformation. The positive yeast transformants were selected and screened for self-activation by testing their growth on media containing inhibitory concentrations of aureobasidin A (AbA). The CDS of *PtrPAT1* was cloned into the pGADT7 vector to generate the prey construct. The prey plasmid was then transformed into the Y1H Gold yeast strains containing the bait constructs, and the transformed cells were cultured on SD/-Ura/−Leu/AbA media. The capacity of transformed yeast cells to propagate on selective media was used as the basis for evaluating protein–DNA interactions.

### Electrophoretic mobility shift assay (EMSA)

The PtrPAT1 CDS was amplified and recombined into the pHMGWA vector, and subsequently expressed in *Escherichia coli* Rosetta (DE3) cells at 37°C for 4 h. The resulting recombinant proteins were then isolated and purified by ProteinIso® Ni-NTA Resin (TransGen Biotech). Oligonucleotides comprising either native or mutated TTTCATGT motif were synthesized, derived from the P/mP sequences, and labeled with cyanine 3 at the 5′-end, serving as probes. EMSA was performed in accordance with established methods [52].

### Dual luciferase assays

Dual LUC assays were performed using previous methods [30]. The *PtrBADH-l* promoter P fragment and its mutated version (mP) were integrated into the pGreenII0800 vector, while PtrPAT1 CDS was incorporated into the pGreenII 62-SK vector to form an effector construct. The reporter and effector plasmids, coupled with the pSoup helper plasmid, were jointly delivered into *A. tumefaciens* for infiltration into *N. benthamiana* leaves. The activities of firefly LUC and *Renilla* luciferase (REN) were measured using the Dual-Luciferase® Reporter Assay System (Promega, USA) on a Tecan Infinite 200 Pro microplate reader to assess transient expression levels. Each sample was analyzed using four independent biological replicates.

### Cold tolerance assay

Cultivation of both T_2_ transgenic tobacco lines and WT seeds was performed on moistened filter paper, adhering to a 16-h light and 8-h dark photoperiod, in a temperature-controlled environment of 25°C. After 3 days, these seedlings were transplanted into pots containing soil and maintained in a growth chamber under the same photoperiod at ambient conditions. For cold treatment, 6-week-old plants were subjected to 0°C for 5 h, followed by a 24-h recovery period at 25°C. To evaluate cold tolerance in trifoliate orange, 1.5-month-old TRV and TRV-PtrPAT1 seedlings were exposed to 0°C for 48 h. The growth of these plants was evaluated before and after cold treatment, with an emphasis on calculating survival rates in tobacco plants after the recovery period. After cold treatment, leaves were harvested for physiological analyses and gene expression profiling.

### Histochemical staining for H_2_O_2_ and O_2_^–^ localization

Localization of H_2_O_2_ and O_2_^·–^ was investigated by *in situ* histochemical staining using 3,3′-diaminobenzidine (DAB) and nitro blue tetrazolium (NBT), respectively. Leaves were immersed in freshly prepared solutions containing 1 mg/mL DAB (in 50 mM potassium phosphate buffer [PPB], pH 3.8) or NBT (in 50 mM PPB, pH 7.8). After a 24-h incubation in the dark at room temperature, the leaf tissues were soaked in 75% ethanol solution at 65°C to allow chlorophyll to fade before being photographed.

### Physiological analyses, determination of GB content, and BADH activity

MDA levels and the activities of antioxidant enzymes, including POD and SOD, were assessed using commercially available kits (BC0025 for MDA, BC0095 for POD, and BC0175 for SOD; Solarbio, China) following the manufacturer’s protocols. Subsequently, 0.2 g of frozen leaf tissues was homogenized in 2 ml of 0.1 mM phosphate-buffered saline, pH 7.8. The resulting mixture was centrifuged at 8000×*g* for 10 min at 4°C. The supernatant was used for further analytical procedures. BADH activity and GB content were measured by enzyme-linked immunosorbent assay following our previous report [30].

### Statistical analysis

The cold treatment procedure was implemented at least twice, each time containing three replicates per line. The statistical evaluation was performed using SPSS 17.0, and inter-group significant differences were determined by one-way analysis of variance by Fisher’s LSD test. All data are shown as mean ± standard error (SE). The statistical significance levels were defined as *P* < 0.05 (*), *P* < 0.01 (**), and *P* < 0.001 (***).

## Supplementary Material

Web_Material_uhae296

## Data Availability

The supporting data for this article is accessible within the article's text and its accompanying online supplementary resources.
