# Supplementary material for: The GRAS transcription factor PtrPAT1 of Poncirus trifoliata functions in cold tolerance and modulates glycine betaine content by regulating the BADH-like gene
Source: Hortic Res. 2024 Oct 23;12(1):uhae296. doi: 10.1093/hr/uhae296 (PMC11775594; doi:10.1093/hr/uhae296)
Supplement: Web_Material_uhae296 [file web_material_uhae296.zip › Supplement data-HR.docx]

SUPPORTING INFORMATION

Article title: The GRAS transcription factor PtrPAT1 of *Poncirus trifoliata* functions in cold tolerance and modulates glycine betaine content by regulating the *BADH-like* gene

Ruhong Ming^1#^, Tian Fang^2#^, Wei Ling^1^, Jingjing Geng^3^, Jing Qu^2^, Yu Zhang^4^, Jianhua Chen^1^, Shaochang Yao^1^, Liangbo Li^1^, Ding Huang^1^*, Ji-Hong Liu^2^*

The following Supporting Information is available for this article:

**Figure S1. Genetic transformation process of tobacco plants overexpressing PtrPAT1.**

**Figure S2. Molecular identification of transgenic tobacco plants overexpressing PtrPAT1.**

**Figure S3. Molecular identification of PtrPAT1 VIGS plants.**

**Figure S4. Relative expression analysis of *PtrBADH-l*, *PtrPOD* and *PtrSOD* in positive PtrPAT1 VIGS plants by qPCR.**

**Table S1. List of primers used in this study.**


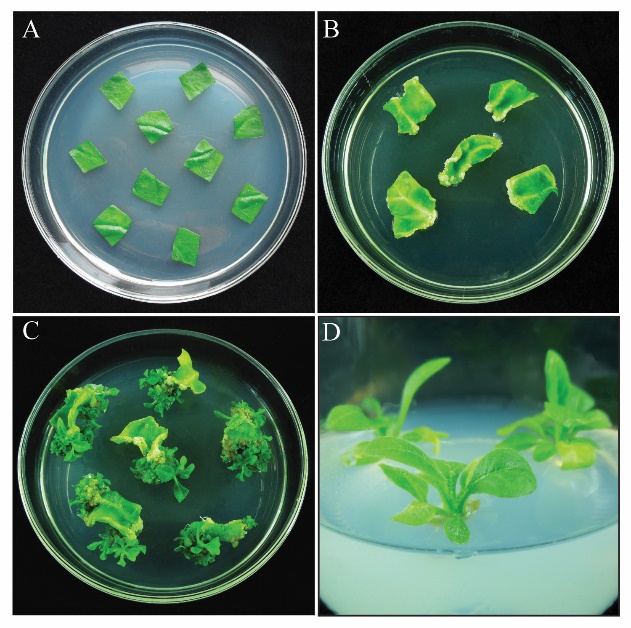


**Figure S1. Genetic transformation process of tobacco plants overexpressing *PtrPAT1*.** (A) Co-culture of tobacco leaf discs. (B) Growth status on the selected medium for one month. (C) Regeneration buds. (D) Rooted plant.


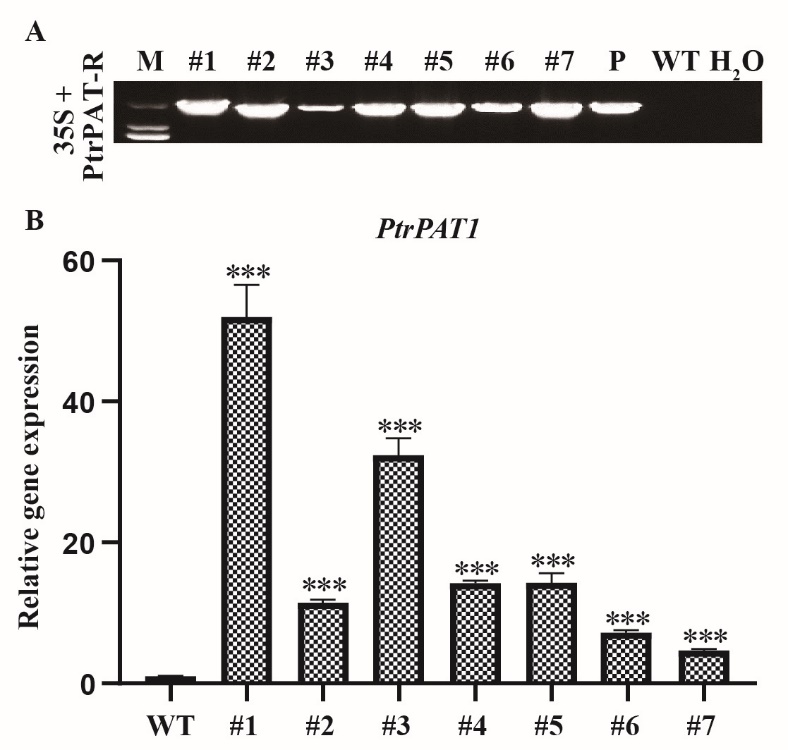


**Figure S2. Molecular identification of transgenic tobacco plants overexpressing *PtrPAT1*.** (A) PCR identification of the transgenic lines using CaMV35S-PtrPAT1 primers. M: DNA Marker; P: positive plasmid; WT: wild type; (B) Expression analysis of PtrPAT1 in positive transgenic lines by qRT-PCR. Ubiquitin was used as internal controls. Data are mean ± SD (n = 3).


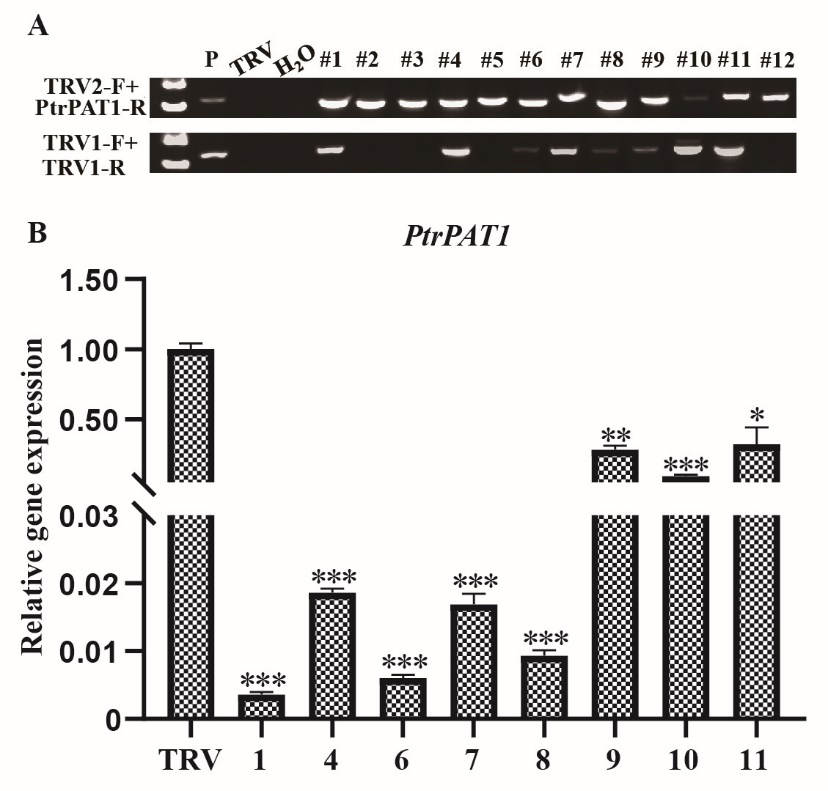


**Figure S3. Molecular identification of PtrPAT1 VIGS plants.** (A) PCR identification of the transgenic lines using two specific primers. Relative expression (B) Gene expression analysis of PtrPAT1 by qPCR in positive TRV-PtrPAT1 plants. WT (wild-type). β-ACTIN was used as an internal control. Data are mean ± SD (n = 3).


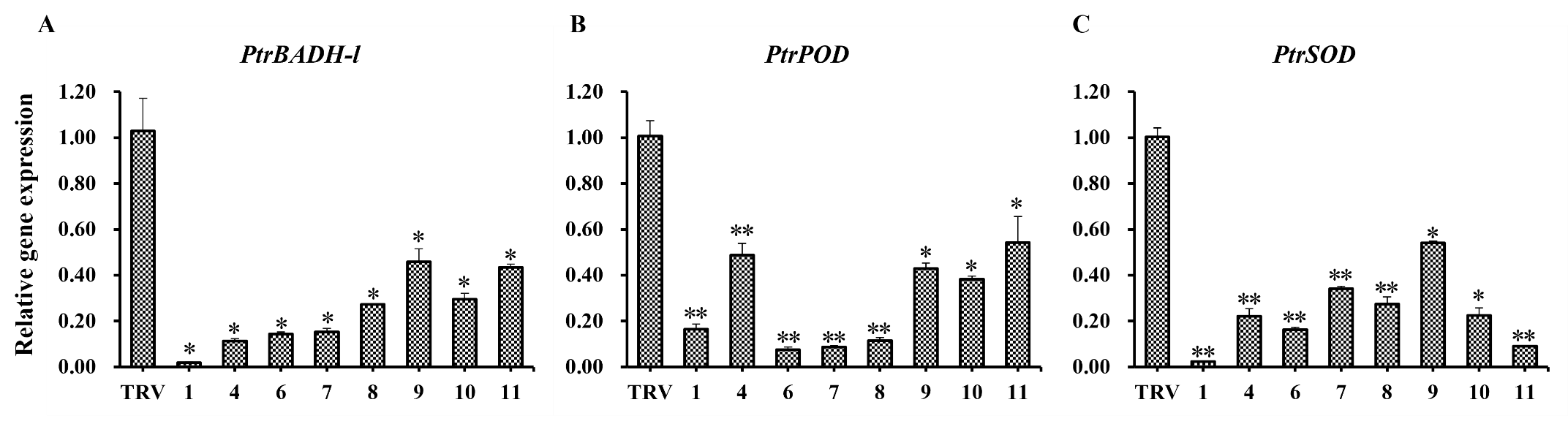


**Figure S4. Relative expression analysis of *PtrBADH-l* (A), *PtrPOD* (B) and *PtrSOD* (C) in positive PtrPAT1 VIGS plants by qPCR.** TRV: control. The β-ACTIN gene was used as an internal control. Data are mean ± SD (n = 3).

**Table S1. List of primers used in this study.**

| **Primer name** | **Sequences (5’ to 3’)** | **Function** |
| --- | --- | --- |
| PtrPAT1-F(XbaI) | GCTCTAGAATGGACTCTCGCCAGATTATTGG | **Gene cloning and positive identification** |
| PtrPAT1-R(BamHI) | CGGGATCCTCAGTGCCAAGCAGAAGCAGATAC |  |
| 35S-F | GCGGATAACAATTTCACACA |  |
| pTRV2-PtrPAT1-F(BamHI) | AGAAGGCCTCCATGGGGATCCCAGTTAAGCCCTGCTGTGGA |  |
| pTRV2-PtrPAT1-R(SmaI) | TGTCTTCGGGACATGCCCGGGCCCTGCAGAGATGATTCCCC |  |
| TRV2-F | ATTCACTGGGAGATGATACGCT |  |
| TRV2-R | AGTCGGCCAAACGCCGATCTCA |  |
| TRV1-F | ATTGAGGCGAATACGATGG |  |
| TRV1-R | ACACCTACGTGTGACACCAACCATG |  |
| PtrPAT1 -ex-F | CAGTTAAGCCCTGCTGTGGA | **Gene expression** |
| PtrPAT1-ex-R | TTGCGGCTTACTACTTCCCC |  |
| PtrBADH-l-ex-F | GATGACGGAAACCAAACGCC |  |
| PtrBADH-l-ex-R | ACGCGATAATGCAGGGAAGT |  |
| Actin-ex-F | CCGACCGTATGAGCAAGGAAA |  |
| Actin-ex-R | TTCCTGTGGACAATGGATGGA |  |
| Ubiquitin-ex-F | GGTGTTTCCAGTGGCGGACG |  |
| Ubiquitin-ex-R | TCCTCCCCTCAGCTACGGGGTAT |  |
| PtrPOD-ex-F | AAGTTGAATTGGGGAGGCGT |  |
| PtrPOD-ex-R | ATCGTATGCGCGCCTGATAA |  |
| PtrSOD-ex-F | AAGTTGAATTGGGGAGGCGT |  |
| PtrSOD-ex-R | GACGCCTCGAATTCTCCACT |  |
| PtrPAT1-101LYFP-F(EcoRI) | ATGGGATCTACTAGTGAATTCATGGACTCTCGCCAGATTATTGG | **Subcellular localization** |
| PtrPAT1-101LYFP-R (BamHI) | GGGGGTACCGTCGACGGATCCGTGCCAAGCAGAAGCAGATAC |  |
| pGBKT7-PtrPAT1-F (EcoR I) | ATGGCCATGGAGGCCGAATTCATGGACTCTCGCCAGAT | **Transcriptional activation activity assays** |
| pGBKT7-PtrPAT1-R (Sal I) | TGCGGCCGCTGCAGGTCGACTCAGTGCCAAGCAGAAGC |  |
| pGBKT7-PtrPAT1-ΔC-F (EcoR I) | ATGGCCATGGAGGCCGAATTCATGGACTCTCGCCAGAT |  |
| pGBKT7-PtrPAT1-ΔC-R (Sal I) | TGCGGCCGCTGCAG GTCGACATTGCCTGATGGAAAACCCTG |  |
| pGBKT7-PtrPAT1-ΔN-F (EcoR I) | ATGGCCATGGAGGCCGAATTCCTGAAGCAGTTATTAATTGCATGC |  |
| pGBKT7-PtrPAT1-ΔN-R (Sal I) | TGCGGCCGCTGCAGGTCGACTCAGTGCCAAGCAGAAGC |  |
| pGADT7-PtrPAT1-F (EcoRI) | GCCATGGAGGCCAGTGAATTCATGGACTCTCGCCAGAT | **Y1H assay** |
| pGADT7-PtrPAT1-R (BamHI) | CAGCTCGAGCTCGATGGATCCTCAGTGCCAAGCAGAAGC |  |
| pAbAi-proPtrBADH-l-full-F (KpnI) | CTTGAATTCGAGCTCGGTACCTGCTTCAACTCAATCGGCTTGG |  |
| pAbAi-proPtrBADH-l-full-R (XhoI) | ATACAGAGCACATGCCTCGAGCGTTTTTTATTTTGCAGAGCAAGG |  |
| pAbAi-proPtrBADH-l-P/mP-F(KpnI) | CTTGAATTCGAGCTCGGTACCGTGTGGGTACACACA |  |
| pAbAi-proPtrBADH-l-P/mP-R (XhoI) | ATACAGAGCACATGCCTCGAGACCATATAGCTAGAGC |  |
| pDONR221-PtrPAT1-F | GGGGACAAGTTTGTACAAAAAAGCAGGCTCCATGGACTCTCGCCAGAT | **EMSA assay** |
| pDONR221-PtrPAT1-R | GGGGACCACTTTGTACAAGAAAGCTGGGTCTCAGTGCCAAGCAGAAGC |  |
| Cy3-P-probe-F (5’) | GAATGAACTATTCATTTTTCATGTTGAATTAGAGTCTCA |  |
| Cy3-mP-probe-F (5’) | GAATGAACTATTCATTTTTttataTGAATTAGAGTCTCA |  |
| mP-probe-R | TGAGACTCTAATTCATATAAAAAAATGAATAGTTCATTC |  |
| P-probe-F | GAATGAACTATTCATTTTTCATGTTGAATTAGAGTCTCA |  |
| P-probe-R | TGAGACTCTAATTCAACATGAAAAATGAATAGTTCATTC |  |
| 62-SK-PtrPAT1-F (BamHI) | CGCTCTAGAACTAGTGGATCCATGGACTCTCGCCAGAT | **LUC assay** |
| 62-SK-PtrPAT1-R (EcoRI) | GATAAGCTTGATATCGAATTCTCAGTGCCAAGCAGAAGC |  |
| LUC-proPtrBADH-l-P/mP-F (KpnI) | GTCGACGGTATCGATAAGCTTGTGTGGGTACACACA |  |
| LUC- proPtrBADH-l-P/mP-R (XhoI) | CGCTCTAGAACTAGTGGATCCACCATATAGCTAGAGC |  |
